# Technical Model of Micro Electrical Discharge Machining (EDM) Milling Suitable for Bottom Grooved Micromixer Design Optimization

**DOI:** 10.3390/mi11060594

**Published:** 2020-06-16

**Authors:** Izidor Sabotin, Gianluca Tristo, Joško Valentinčič

**Affiliations:** 1 Faculty of Mechanical Engineering, University of Ljubljana, Aškerčeva 6, 1000 Ljubljana, Slovenia; izidor.sabotin@fs.uni-lj.si; 2 Department of Industrial Engineering, University of Padua, Via Gradenigo, 6/a, 35131 Padua, Italy; gianluca.tristo@unipd.it; 3 Chair of Micro Process Engineering and Technology (COMPETE), University of Ljubljana, Večna pot 113, 1000 Ljubljana, Slovenia

**Keywords:** micromachining, micro EDM milling, empirical modelling, micromixer, design for manufacturing, computational fluid dynamics

## Abstract

In this paper, development of a technical model of micro Electrical Discharge Machining in milling configuration (EDM milling) is presented. The input to the model is a parametrically presented feature geometry and the output is a feature machining time. To model key factors influencing feature machining time, an experimental campaign by machining various microgrooves into corrosive resistant steel was executed. The following parameters were investigated: electrode dressing time, material removal rate, electrode wear, electrode wear control time and machining strategy. The technology data and knowledge base were constructed using data obtained experimentally. The model is applicable for groove-like features, commonly applied in bottom grooved micromixers (BGMs), with widths from 40 to 120 µm and depths up to 100 µm. The optimization of a BGM geometry is presented as a case study of the model usage. The mixing performances of various micromixer designs, compliant with micro EDM milling technology, were evaluated using computational fluid dynamics modelling. The results show that slanted groove micromixer is a favourable design to be implemented when micro EDM milling technology is applied. The presented technical model provides an efficient design optimization tool and, thus, aims to be used by a microfluidic design engineer.

## 1. Introduction

Micromachining plays a crucial role in bringing new chemical, medical, optical, automotive and semiconductor applications and products to market [1,2]. The industry strives for shorter cycle times to minimize the manufacturing costs. The research field that largely benefited from microengineering technologies (MET) development is microreactor technology, which exploits microstructured devices for realization of (bio)chemical processes [3,4].

A microreactor is commonly understood to be a continuous flow reactor which utilizes microchannels and similar micro features in order to manipulate flow of reactants. The basic configuration of a microreactor consists of a micromixer, a reaction unit and a separator. The micromixer, as a crucial functional part of the microreactor with the task of mixing reactants, usually represents the most challenging geometry to be machined. 

**Figure 1:**
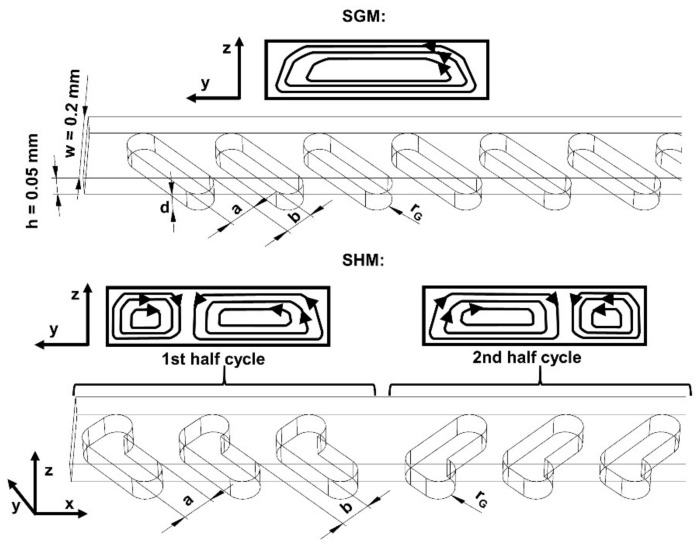
In the upper part, a slanted groove micromixer (SGM) geometry with schematic presentation of path lines in the channel cross-section is depicted. In the lower part, a staggered herringbone micromixer (SHM) geometry with schematic presentation of path lines in the cross-section for each half-cycle is depicted. Denotations: *h*—channel height, *w*—channel width, *d—*groove depth, *a*—groove width, *b*—ridge width, and *r*_G_—groove corner rounding radius.

One of the established micromixer designs is the bottom grooved micromixer (BGM), first introduced by Stroock et al [5] in 2002. The working principle of the BGM is based on the grooves embedded in the bottom of the microchannel which laterally transport fluid fractions and cause lateral circulation. This circulation motion promotes mixing by stretching and folding the fluid along the microchannel [5,6]. Generally, there are two basic designs of BGMs, namely, slanted groove micromixer (SGM) and staggered herringbone micromixer (SHM) (Figure 1). Grooves in SGM design are inclined at an angle with respect to the axis along the channel, whereas grooves in SHMs are the shape of a herringbone pattern. Many publications dealing with BGMs geometry optimization to enhance mixing exist, the most notable being by: Yang et al. [7], who applied the Taguchi method; Aubin et al. [8], who analysed BGM design using spatial data statistics and the maximum striation thickness parameter; Ansari and Kim [9], who applied the radial basis neural network method; Lynn and Dandy [6], who investigated helical flows; Williams et al. [10], who developed the analytical model of mixing; Cortes-Quiroz et al. [11], who used design of experiments, the function approximation technique and the multi-objective genetic algorithm; and Hossain et al. [12], who investigated grooves embedded in the top and bottom walls. The topic of BGM design optimization is highly relevant up to the present time, as demonstrated in very recent publications [13,14]. Thus, optimal design of a BGM has yet to be discovered with respect to specificities of its application and taking into account applied micromanufacturing technology and substrate material.

Traditionally, BGMs [5,10,15] and microfluidic platforms with similar geometrical features such as micro heat exchangers [16,17] and fuel cell components [18] are machined in silicon, thus, utilizing microfabrication technologies (i.e., various lithographic processes). On the other hand, metallic materials are gaining substantial importance in microfluidic devices and micro-electro-mechanical systems (MEMS) for their ability to withstand high pressures, harsh environmental conditions and chemically reactive conditions [19]. In this respect, stainless steel substrates offer significant advantages over silicon: excellent mechanical, electrical, thermal and chemical properties, and they are easy to clean, thus, they are reusable mostly due to material robustness [20,21,22]. Accordingly, micro EDM milling is a versatile and well established micromachining process which is readily capable of machining geometries applied in aforementioned applications [23].

Micro EDM milling is a thermal (or energy-assisted) process for contactless material removal of electrically conductive materials where a rotating cylindrical electrode removes material layer by layer with kinematics similar to those of conventional milling. Material is removed due to local melting and evaporation caused by a sequence of electrical discharges occurring in an electrically insulated gap between the tool electrode and the workpiece. Technology advantage stems from being able to machine complex shapes with high precision regardless of material hardness.

To date, a generally valid physical model of EDM material removal does not exist and there is a lack of a direct link between theoretical models of material removal mechanisms and industrial applications [24]. In the literature, many micro EDM milling modelling approaches were executed, such as mathematical modelling [25], cellular automata approach [26,27], non-uniform rational basis spline (NURBS) surface warping [28] and many others thoroughly reviewed in [29]. These modelling approaches yield valuable information in the context of understanding the underlying physical principles of the technology and can be applied for its advancements. However, in the view of process selection and industrial applicability, simple technical models can yield enough information to the designing engineer for the purpose of product design optimization in line with the design for manufacturing (DFM) paradigm [30]. By incorporating a few simple empirical equations encompassing the main technology characteristics, the technical model can be used as a tool by the design engineer, who does not need a thorough in-depth knowledge about the technology, for designing a functional product with lower machining costs.

For this reason, a simple technical model of micro EDM milling is constructed and presented in this paper. Main relations specific to micro EDM milling technology were obtained using empirical modelling. The output of the model is the total machining time of a single feature to be machined, which is determined by the contribution of duration of three operations, namely electrode dressing time, electrode wear control time and actual feature erosion time. The feature geometries are parametrically presented as an input to the technical model. One of the aims of this paper is to further familiarize the microfluidic research community with the micro EDM milling technology and its aspects on designing a microfluidic device. Demonstration of the technical model usage is presented through optimization of a BGM design. The developed technical model presents a simple platform, the applicability of which could be further expanded to a wider range of microfeatures by upgrading the technology data and knowledge base (DKB). At present, its applicability is confined to groove microfeatures which are commonly applied in BGMs; however, similar repetitive geometries are applied in various microfluidic applications [17,31,32]. The novelty of this work is in construction of an easy to understand technical model that can be used by a non-expert for microfluidic design optimization. Additionally, the results of intertwining the technical and computational fluid dynamics modelling give novel insights on the influence of geometrical parameters on the mixing performance of the BGM.

## 2. Materials and Methods

### 2.1. Investigation Workflow

**Figure 2:**
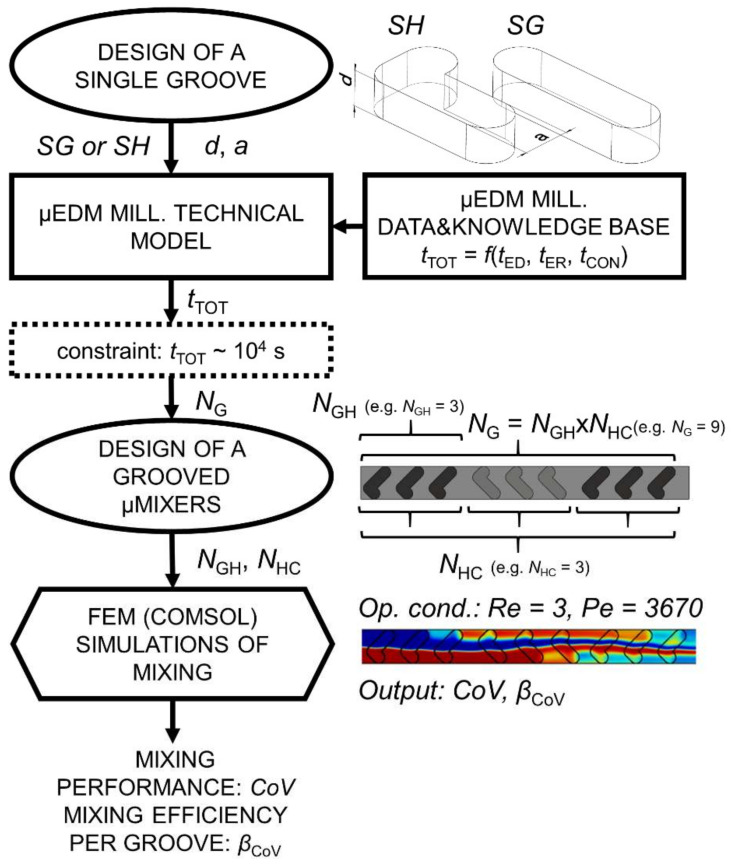
Investigation workflow. Denotations: *SH*—staggered herringbone groove design, *SG*—slanted groove design, *d*—groove depth, *a*—groove width, *t*_ED_—electrode dressing time, *t*_ER_—erosion time, *t*_CON_—electrode wear control time, *t*_TOT_—overall machining time, *N*_G_—number of grooves in a configuration, *N*_GH_—number of grooves in a half-cycle, *N*_HC_—number of half-cycles, *Re*—Reynolds number, *Pe*—Peclet number, *CoV*—mixing coefficient of variance, and *β*_CoV_—mixing efficiency per groove.

The workflow of this investigation is presented in Figure 2. The mixing efficiency of the BGM is critically dependent on the geometry of one groove, which is defined by its width and depth, its basic shape, namely slanted (SG) or staggered herringbone (SH), and their number and orientation in a mixer configuration (see also Section 2.2). The defined single groove design presents the main input to the micro EDM milling technical model. Output of the technical model is the overall machining time *t*_TOT_ for the input geometry and, by defining a time constraint, results in the number of grooves (*N*_G_) for the whole BGM configuration that can be machined in the given time. The machining time of the main microchannel is included in the time constraint as well. The technical model is supported by the micro EDM milling DKB, which was constructed based on the experimental approach (see Section 2.3). After determining *N*_G_, permutations of the groove sequence layout were manually determined, resulting in BGM configurations with a different number of half-cycles (*N*_HC_) and number of grooves in one half-cycle (*N*_GH_). Mixing performances of adequate designs were determined by simulations using the numerical finite element software COMSOL (5.0, COMSOL, Inc., Burlington, MA, USA).

### 2.2. BGM Geometry and Mixing Simulation Tool

For the purpose of this investigation, the microchannel is oriented along the *x*-axis and its cross-section is fixed to width *w* = 200 µm and height *h* = 50 µm (Figure 1). This cross-section dimension is typical for generic microfluidic chips [33] and, due to its low aspect ratio (*h*/*w* = 0.25), it narrows the residence time distribution and reduces axial dispersion of the fluid species [34]. The grooves are inclined at a 45° angle to the *x*-axis and asymmetry of the SH grooves apex position was fixed to 1/3 x *w*, since this was found to be the optimal groove geometry [5,6,7]. Groove width *a* varied between 48 µm and 106 µm and groove depth *d* between 40 and 75 µm. The maximum aspect ratio of the grooves (*d*/*a*) incorporated in micromixer configurations was limited to 0.85 due to the possibility of air entrapment within deeper grooves [35] and to avoid dead volumes. The minimum groove width of 48 µm was selected since it is a typical width for micromixers produced by soft lithography and can be stably machined by micro EDM milling with a ~40 µm thick electrode. The upper boundary for groove width *a* is defined by the width of the channel *w*, since wider grooves machined with a circular electrode would be more similar to a circular blind hole than a groove. The rounding of the groove corners *r*_G_ corresponds to the smallest electrode diameter used for its machining and *r*_G_ varied between 25 and 53 µm. Ridge length *b* between consecutive grooves, being one of the least influential geometric parameters [7,36], was fixed to 70 µm. The geometrical parameters of simulated groove designs are gathered in Table 1.

Mixing performances of BGM configurations compliant with micro EDM milling technology were evaluated by computational fluid dynamics (CFD) modelling using COMSOL Multiphysics 5.0 software. The software implements the finite element method to numerically solve governing equations. Since the applied simulation software settings are described in detail in [36], only a condensed description is given here. The flow field at steady state was calculated by solving Navier–Stokes (NS) equations for an incompressible fluid, and mass transport of dissolved species was numerically calculated by solving convection–diffusion (CD) equations. Since numerical simulations of mixing are prone to artificial diffusion of species [6,37], the simulation tool setup was verified against experimentally obtained results presented in [5,10], where confocal microscopy was applied to obtain precise spatial mixing profiles. Both quantitative (coefficient of variance) and qualitative (concentration profiles in the channel cross-section) results showed excellent agreement up to the Péclet number (*Pe*) of 6250, thus, we considered numerical model setup to be adequate.

Mixing performance was evaluated by applying coefficient of variance *CoV* as recommended in [38], defined by:

(1) $$CoV=\frac{\sqrt{1/N\sum_{n=1}^{N}{\left(c_{n}-\bar{c}\right)}^{2}}}{\bar{c}}$$

where *CoV* denotes the coefficient of variance in the *yz*-cross-section plane at the end of the channel, *c*_n_ is the concentration at a point in the cross-section plane, *N* is the number of concentration points in the plane (exported in a uniform grid with 2.5 µm spacing resulting in *N* = 1600), and $\bar{c}$ denotes complete mixing. A value of *CoV* closer to 0 corresponds to better mixing performance.

To take into account the geometrical parameter of the number of grooves *N*_G_ per BGM configuration, an additional parameter interpreted as mixing efficiency per groove was estimated as:

(2) $$\beta_{\mathrm{CoV}}=1/\left(CoV\cdot N_{G}\right)$$

where a higher *β*_CoV_ value corresponds to a higher mixing efficiency of BGM configuration.

All further simulations were performed using the following conditions: the working fluid was water at 20 °C, the solute diffusion coefficient *D* = 10^−9^ m^2^/s, the average inflow velocity *v* = 37.8 mm/s corresponding to the Reynolds number of 3 and *Pe* = 3670, the boundary condition at outflow set to 0 Pa (pressure, no viscous stress), no-slip condition at walls, fluid concentration in one half of the channel of 1 mol/m^3^ (color coded red) and the other half of 0 mol/m^3^ (color coded blue). Data visualizations and post-processing of simulation results were done using associated functions in COMSOL and MATLAB (R2019b, MathWorks Inc., MA, USA).

### 2.3. Micro EDM Milling

**Figure 3:**
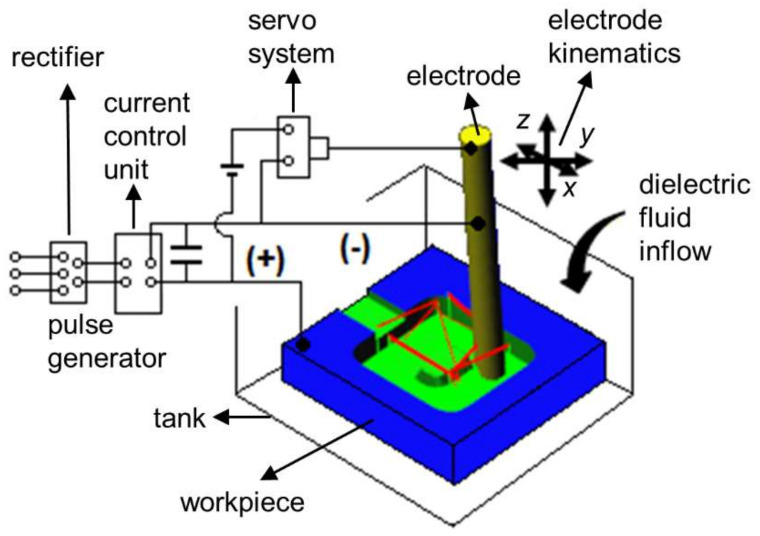
Schematic presentation of the micro EDM milling machine.

The technical model is based on experimental data obtained on Sarix SX-200 micro EDM milling machine (SARIX SA, Sant’Antonino, Switzerland), thus, the set-up parameters, also referred to as the machining regime, are specific to the used machine (further elaborated in Section 2.3.2). Input parameters encompass the workpiece, electrode, machining strategy, dielectric fluid and machining parameters. The latter define the settings of the pulse generator and the electrode feeding system. For clearer presentation of the micro EDM milling working principle, a diagram is shown in Figure 3.

**Figure 4:**
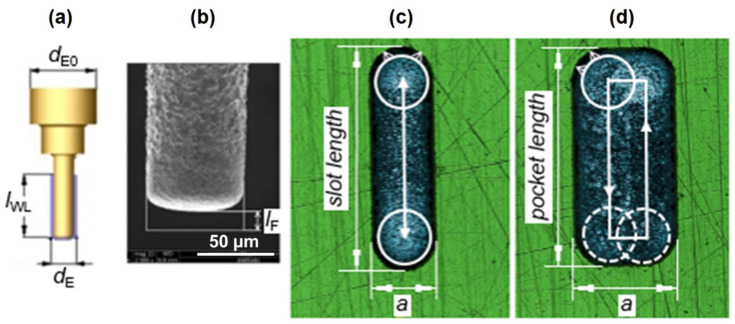
(**a**) Schematic presentation of the dressed electrode used for micro EDM milling with its main features: *d*_E0_—diameter of primary electrode, *d*_E_—electrode working diameter, *l*_WL_—electrode working length. (**b**) A scanning electron microscope (FEI Quanta 400, FEI, Hillsboro, Ore., USA) image of a worn electrode where *l*_F_ denotes linear wear. (**c**) A confocal microscope image (Sensofar PLµ, NEOX, Sensofar Medical, Terrassa, Spain) of a groove (*a* = 90 µm) presenting slotting strategy for machining. The white circles represent the cross-section of the electrode and the arrows at the circle’s circumference denote the sparking gap. (**d**) Presentation of pocketing strategy for machining of a pocket groove in 2 passes (*a* = 140 µm).

Thin cylindrical tungsten carbide rods with initial diameter *d*_E0_ of 300 µm were used as tool electrodes. The machine was equipped with a wire dressing unit (ARIANNE) and a laser scan micrometer Mitutoyo LSM-500s (Mitutoyo Corporation, Kawasaki, Kanagawa, Japan), which monitors the reduced diameter (*d*_E_) of dressed electrodes (Figure 4a). By means of mentioned integrated components, the initial electrode is grinded down to the desired diameter, which depends on the width of the geometry to be machined and the spark gap. Slotting and pocketing machining strategies were considered (Figure 4c,d). Hydrocarbon oil was used as a dielectric fluid (HEDMA 111). All experiments to acquire micro EDM milling DKB were performed on corrosive resistant steel X2CrNiMo17-12-2 (Acroni Ltd., Jesenice, Slovenia), which is commonly used in chemical and pharmaceutical industry. Prior to machining, the steel workpieces were ground on both sides in order to establish parallel surfaces. In the next step, the surfaces were hand polished to achieve a mirror-like surface finish (roughness of *Ra* ~ 0.05 µm, measured with MARSURF PS 10 profilometer (Mahr GmbH, Göttingen, Germany). Due to imperfect polishing, minor surface scratches can be noticed on the confocal microscope images of the grooves (Figure 4c,d). However, these scratches were small enough and have insignificant influence on the obtained machining results.

Although the technical model built within this investigation is based on the specific machine and material used, the generalized findings can also be applied when using other micro EDM milling systems in the view of part design optimization for manufacturing.

In general, the aim of all machining processes is to produce parts with the desired geometrical accuracy and surface integrity. In micro EDM milling, accuracy greatly depends on compensation of the tool electrode wear [39,40,41,42]. SX-200 implements the linear tool wear compensation method (LCM) [43]. Electrode wear is often measured with linear wear *l*_F_ (Figure 4b), which defines the coefficient of linear wear $\vartheta_{F}$ by dividing the *l*_F_ with erosion time *t*_ER_ [44].

In micro EDM milling, the machining is done in a sequence of 3 machining operations:

1. op_1_: dressing of the electrode using on the machine integrated wire dressing unit. Time needed for this operation is denoted as *t*_ED_ and is a function of:*t*_ED_ = *f*(*d*_E_(geometry, gap(regime)), electrode working length($\vartheta_{F}$*,* regime));
2. op_2_: actual erosion time *t*_ER_ = *f*(*d*_E_, regime, strategy, *MRR,* volume of geometry);
3. op_3_: time for electrode wear control *t*_CON_ = *f* (depth of the geometry, $\vartheta_{F}$).

To achieve the desired tolerances of the machined geometries (in our case, within ±4 µm), all three operations are executed every time when a new feature is being machined. Thus, the feature machining time is a sum of all three operations.

#### 2.3.1. Dressing of the Tool Electrode

Electrode dressing time *t*_ED_ was investigated with respect to electrode nominal diameter *d*_E_ and electrode working length *l*_WL_ (see Figure 4a). These two parameters, in combination with starting electrode diameter *d*_E0_, determine the shape and dressing time of the tool electrode. The electrode *l*_WL_ defines the maximum depth of the slot that could be potentially machined considering no electrode wear. Default factory settings for the on-machine wire dressing unit were used. Investigated *d*_E_ range was from 40 to 120 µm. The electrode diameter tolerance was set to ±4 µm. In the first part of experiments, *l*_WL_ was fixed at 150 µm. For investigation of the influence of *l*_WL_ on *t*_ED_, the length of *l*_WL_ was fixed to 8 × *d*_E_, which is the maximum length ratio recommended by the machine tool manufacturer. For each *d*_E_, electrode dressing was repeated 4 times to estimate the electrode dressing time *t*_ED_. Altogether, 80 experiments on electrode dressing were performed.

#### 2.3.2. Material Removal Rate and Erosion Time

Due to required low roughness (*Ra* ~ 0.5 µm), the finishing machining regime suggested by the Esprit CAD/CAM system suitable for stainless steel was used (Table 2). The same machining regime is suggested for electrode diameters between 50 and 120 µm. For *d*_E_ = 40 µm, the system suggests finer settings with lower pulse current and voltage. Machining parameters applied for the erosion of the main channel (*d*_E_ = 183 µm) are also presented.

**Figure 5:**
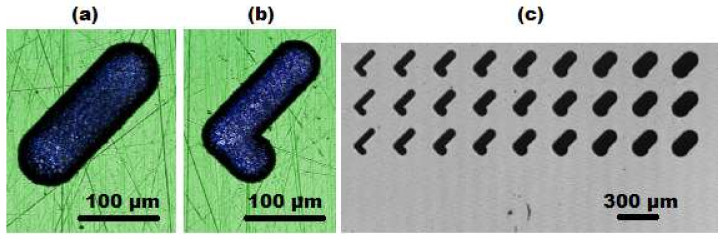
Microscope images of *SG* and *SH* slots, which can be implemented in the *w* = 200 µm wide microchannel. (**a**) *SG* slot (*d*_E_ = 75 µm, *d* = 80 µm) and (**b**) *SH* slot (*d*_E_ = 60 µm, *d* = 80 µm). (**c**) *SH* slots (*d*_E_ = 40–120 µm, *d* = 80 µm).

**Figure 6:**
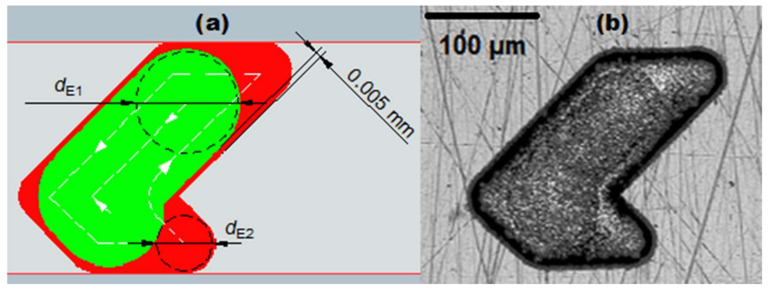
(**a**) Depiction of the 2-electrode strategy for machining of the SH groove. 5 µm band is left by larger electrode *d*_E1_, thus, its diameter is determined by: *d*_E1_ = *a* – 2 × *l*_GAP_ – 2 × 5 µm. (**b**) Confocal microscope image of the SH groove machined with *d*_E1_ = 92 µm and *d*_E2_ = 44 µm (*d* = 100 µm, *a* = 106 µm, *r*_G_ = 25 µm).

Several sets of slots and pockets were machined to determine the influence of *d*_E_ and machining regimes on material removal rate (*MRR*) and electrode wear. Each feature was machined by an electrode producing total width of the feature in one pass (slotting strategy), except in the case of pockets:

- slots: length of 310 µm, *d* = 100 µm, *d*_E_ = 40–120 µm, 2 repetitions (Figure 4c);
- pockets: length of 310 µm, *d* = 100 µm, *d*_E_ = 40–120 µm, 2 passes with 40% overlap, 2 repetitions (Figure 4d);
- *SG* slots: *d* = 80 µm, *d*_E_ = 50, 75, 100 µm, 3 repetitions (Figure 5a);
- *SH* slots: *d* = 80 µm, *d*_E_ = 40–120 µm, 3 repetitions (Figure 5b,c);
- *SH* slots: *d* = 40, 120 µm, *d*_E_ = 40, 70, 100 µm, 1 repetition;
- *SH* groove—2-electrode strategy: *d* = 100 µm, *d*_E1_ = 92 µm, *d*_E2_ = 44 µm, 2 repetitions (Figure 6).

This resulted in 76 machined grooves. Removed workpiece volume (*V*_W_) was determined by measuring groove dimensions with the Sensofar PLµ NEOX confocal microscope (Sensofar Medical, Terrassa, Spain). *MRR* was determined as *V*_W_/*t*_ER_. In all experiments stable machining was observed, thus suitable machining regimes were used. The results were added to DKB.

When groove corner roundings are relatively narrow, a 2-electrode strategy is employed: a wider electrode is applied to remove a larger portion of groove volume and, afterwards, thinner electrode removes material around the edges (Figure 6). The 2-electrode strategy is significantly faster compared to machining the whole groove volume with only one thinner electrode (1-electrode pocketing strategy). The technical model considers both strategies and selects the most appropriate one for the given geometry.

At the end of machining, linear wear *l*_F_ was measured by the touch method. The touch method was implemented on the used machine and consists of traversing the electrode to the reference measuring point. Then, the electrode is gradually approaching the reference point in the *z*-axis. Upon touch, detected by a short circuit signal, the difference in readout of the *z-*coordinate compared to the previous iteration determines the electrode linear wear. For further information about the touch method, see [45]. Volumetric wear ratio ϑ was calculated as [46]:

(3) $$\vartheta =\frac{V_{E}}{V_{W}}=\frac{l_{F}\pi d_{E}^{2}}{4V_{W}},$$

where *V*_E_ denotes volumetric wear of the electrode.

#### 2.3.3. Electrode Wear Control

Compensation of the tool electrode wear during erosion is crucial for the achieved machining accuracy. For this reason, LCM is implemented on the machine, where linear electrode wear is intermittently monitored during erosion after selecting the number of layers by the touch method. Consequently, linear electrode wear is compensated during machining with a gradual movement of the electrode tip position in the *z*-axis. The results of preliminary experiments (groove length = 310 µm, *d* = 100 µm) showed that the control after erosion of every 10 µm in depth is adequate for achieving tolerances in the *z*-axis within ±4 µm. Individual control time (*t*_CON_) comprises of touch method measurement duration and electrode traveling to and from the reference measuring point.

### 2.4. Implementation of the Technical Model

**Figure 7:**
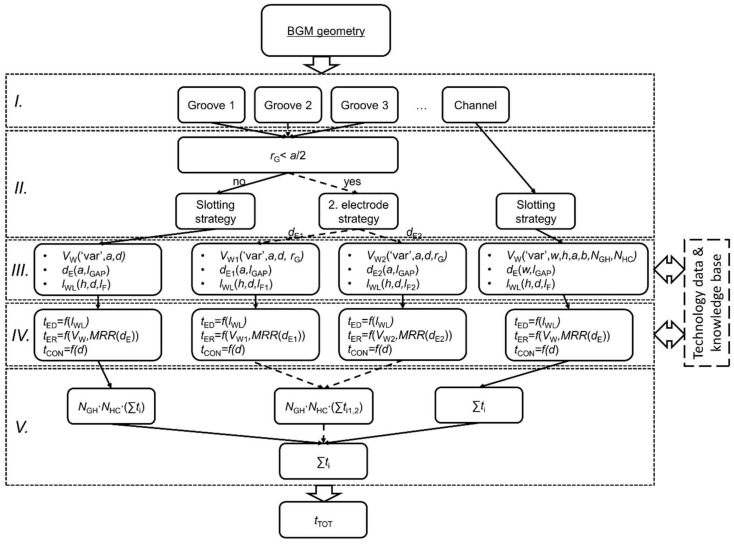
Detailed scheme of the implemented technical model where ‘var’ in dashed box *III.* denotes either SG or SH design.

The technical model was programmed in MATLAB and is schematically presented in Figure 7. The input to the model is parametrically defined BGM geometry, basically determined by the variant (SG or SH) and dimensions of a single groove (step *I*). Based on the groove corner rounding radius (*r*_G_), 2-electrode strategy is selected if *r*_G_ is smaller than *a*/2 (Figure 7, step *II*). In step *III*, groove volume *V*_W_ is calculated. According to groove width *a*, electrode working diameter *d*_E_ is determined by taking into account spark gap (*l*_GAP_) consistent with the applied machining regime. Needed electrode working length *l*_WL_ is determined by the absolute depth of the groove (i.e., sum of *h* and *d*) and corresponding tool wear. In step *IV*, durations of all three machining operations are calculated with respect to relevant relations defined in DKB. In step *V*, the machining time of a single groove is multiplied by the number of grooves in a BGM configuration.

Microchannel machining time is calculated separately and, at fixed width *w* and height *h*, depends only on its length, which is determined by the BGM configuration. The sum of machining times of all features (i.e., grooves and microchannel) defines total machining time *t*_TOT_.

### 2.5. Simulated BGM Designs

Overall, 23 different BGM designs compliant with the dimensional boundaries given in Table 1. were simulated to estimate their mixing performances. A particular BGM design was considered on the criteria of having overall machining time *t*_TOT_ of approximately 10^4^ s, and that the number of half-cycle *N*_HC_ at SH configurations corresponded to an integer. Relaxation of the *t*_TOT_ constraint was made for SH configurations where 2-electrode strategy was applied. Since this strategy requires longer machining time per groove and mixing performance of the SH design is significantly enhanced with the increase of number of half-cycles, the *t*_TOT_ criterion was extended up to 1.5 × 10^4^ s, so that at least 9 grooves were machined (resulting in configuration with *N*_HC_ and *N*_G_ of 3). All configurations are parametrically presented later in Section 3.2 (Table 2).

## 3. Results and Discussion

### 3.1. Micro EDM Milling DKB Construction

**Figure 8:**
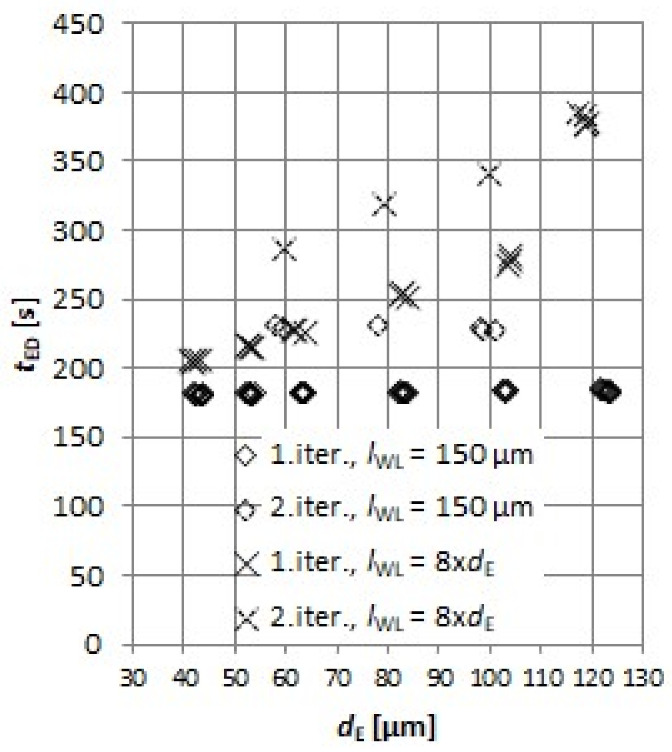
Electrode dressing times *t*_ED_ for various electrode diameters *d*_E_ and electrode working lengths *l*_WL_.

As explained in Section 2.3.1, two sets of experiments were performed to model the relationship defining electrode dressing time *t*_ED_: first set with constant *l*_WL_ and the second set with *l*_WL_ being 8 × *d*_E_. The results show that *t*_ED_ depends on the electrode working length *l*_WL_ and obtained accuracy of the electrode diameter in the first step of the dressing. As evident from Figure 8, the on-machine factory implemented electrode dressing procedure takes about the same amount of time when set to *l*_WL_ = 150 µm regardless of the set *d*_E_: *t*_ED_ = 182 ± 1.5 s. Thus, for the electrodes dressed in the first iteration, *t*_ED_ is influenced only by the electrode working length *l*_WL_.

**Figure 9:**
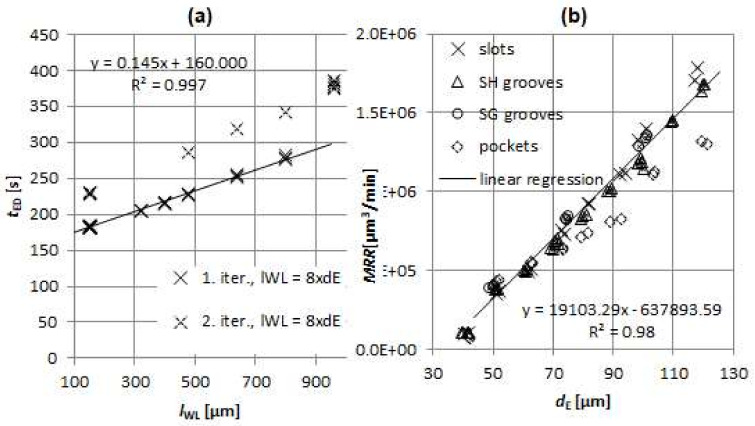
(**a**) Electrode dressing times *t*_ED_ for various electrode working lengths (*l*_WL_ = 8 × *d*_E_). Linear regression was applied to the data set, where the electrodes are dressed in the first iteration. (**b**) Material removal rate (*MRR*) with respect to electrode diameter *d*_E_. For the linear regression, only slotting experiments (slots, SH and SG grooves) were considered.

It should be noted that, in minor cases, the electrode is dressed in second iteration. Namely, if the laser scan measurement of the *d*_E_ after the first iteration of dressing is out of set tolerances, the second iteration of electrode dressing is executed, but this happened randomly and rarely (only in 16% of the cases) and, thus, it is not included in the technical model. *t*_ED_ operation was therefore modelled with linear function (Figure 9a) by applying linear regression resulting in equation

(4) $$t_{\mathrm{ED}}=0.145l_{\mathrm{WL}}+160\;\left[s\right],$$

where the units for *l*_WL_ are in µm and a correlation coefficient of *R*^2^ = 0.99 was obtained.

Investigation of *MRR* relation with respect to *d*_E_ exhibits a linear trend (Figure 9b). For micro EDM milling, it is known that material removal per discharge mainly depends on pulse discharge energy [47]. Since, for most electrode diameters (with exception of *d*_E_ = 40 µm), the same machining regime was used, the *MRR* increased due to the increase of electrode face surface rendering favourable spark conditions. Thus, *MRR*(*d*_E_) was modelled by applying linear regression resulting in equation

(5) $$MRR=19103d_{E}-637893\;\left[µm^{3}/min\right],$$

where the units for *d*_E_ are in µm and correlation coefficient of *R*^2^ = 0.98 was obtained. A plausible explanation for rising linear trend of *MRR*(*d*_E_) is in linear increase of the effective eroding surface of the electrode which is, at constant machining layer depth *z*_1L_, defined by (*d*_E_ + 2*l*_GAP_)∙*z*_1L_ as stated in [43]. With the increase of the active area, the discharges are more sparsely distributed thus, rendering favourable spark conditions. Average gap at groove machining was measured to be *l*_GAP_ = 3 ± 0.3 µm.

*MRR* for pocketing strategy is lower due to the overlap which reduces effective eroding surface of the electrode. Thus, the slotting strategy is favourable in terms of machining BGM designs.

**Figure 10:**
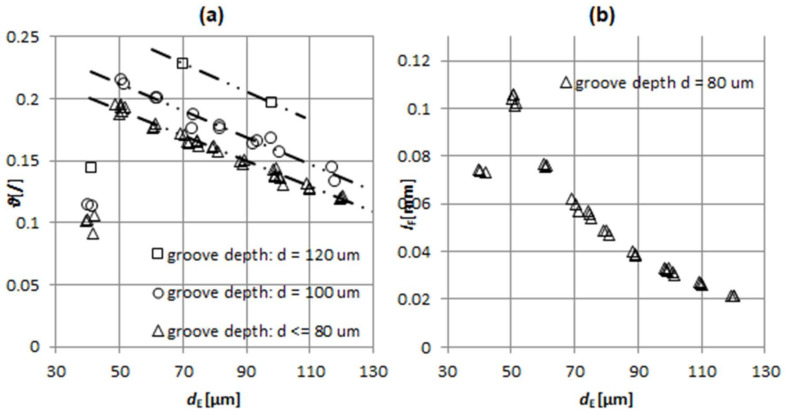
(**a**) Volumetric wear ratio ϑ with respect to electrode working diameter for grooves of different depths. (**b**) Linear wear *l*_F_ with respect to electrode working diameter for groove depths of d = 80 µm.

Electrode tool wear was monitored during machining. It influences machining time *t*_TOT_ via adding a compensation length to *l*_WL_. It can be noted (Figure 10a) that for a fixed groove depth and machining *regime*, the volumetric wear ratio ϑ linearly decreases with increase of *d*_E_. This is due to a higher wear resistance of larger electrodes, since wear is correlated to electrode thermal properties. Thus, larger surface area of the electrode tip, which grows proportionally with $d_{E}^{2}$*,* conducts heat in the electrode bulk and, due to larger electrode volume, the wear caused by melting is reduced. At the same time, discharges are less densely distributed due to the bigger eroding surface. Simultaneously, when slotting with larger electrodes, more dielectric fluid surrounds the electrode within the groove and acts as a coolant. Obtained results are consistent with the findings in [46], which states that larger electrodes are more wear resistant due to their bulk volume and correlated manifestation of thermal erosion caused by sparks.

In the case of machined grooves deeper than 80 µm, ϑ increases by offsetting a linear trend (Figure 10a). A plausible explanation of this observation would be in worse flushing conditions occurring in deeper grooves. This effects electrode wear in two ways. Firstly, the debris within the spark gap start to accumulate, which cause arcing and, thus, promote electrode wear. Secondly, the cooling effect of the dielectric is reduced. Figure 10a also indicates the influence of machining *regime* with lower pulse current and voltage used for *d*_E_ = 0.04. Volumetric wear ration ϑ and linear wear *l*_F_ (Figure 10b) significantly decrease due to less aggressive machining regime.

Stemming from practice and considering the simplicity of the technical model, adding a safety factor of 100% to the highest linear wear recorded (*l*_F_ = 103 µm at *d*_E_ = 50 µm, *d* = 80 µm) was introduced to calculation of electrode working length

(6) $$l_{\mathrm{WL}}=structure\;depth+200\;µm,$$

where structure depth in the case of a micromixer machining represents a sum of main channel height *h* and groove depth *d*.

Analysis of 60 measurements of the time required for control of the electrode linear wear resulted in an average control time ${\bar{t}}_{1\mathrm{CON}}$ of 22.6 s with a standard deviation of 0.9 s. With respect to reasoning described in Section 2.3.3, *t*_CON_ was determined as:

(7) $$t_{\mathrm{CON}}=\frac{d\;\left[µm\right]}{10\;\left[µm\right]}{\bar{t}}_{1\mathrm{CON}}\;\left[s\right].$$

The technical model was verified by machining of test grooves with varying depths and widths. The model prediction of the machining time was within 8% of the measured machining time. With regards to the stochastic nature of the EDM process, we consider that the technical model is adequate for its purpose.

To give a clearer presentation on how the technical model algorithm works, a short manual demonstration of machining time calculation for a single slanted groove is demonstrated. For parametrical input parameters *a* = 88 µm, *r*_G_ = *a*/2, d = 40 µm, *w* = 200 µm and *h* = 50 µm the algorithm works as follows:

- based on the premise *r*_G_ == *a*/2 single electrode slotting strategy is selected;
- volume to be removed is calculated as: $V_{W}=\left(\frac{\pi a^{2}}{4}+\sqrt{2}\cdot a\cdot \left(w-a\right)\right)\times d=8\times 10^{5}{\mu m}^{3};$
- electrode diameter is calculated as: *d*_E_ = *a -* 2∙*l*_GAP_ = 82 µm;
- electrode working length (Equation (6)): *l*_WL_ = *f*(*h,d*) = 290 µm;
- time of electrode dressing (Equation (4)): *t*_ED_ = *f*(*l*_WL_ = 290 µm) = 202 s;
- material removal rate (Equation (5)): *MRR* = *f*(*d*_E_ = 82 µm) = 9.3 × 10^5^ µm^3^/min;
- erosion time: *t*_ER_ = *V*_W_/*MRR* = 51 s;
- control time (Equation (7)): *t*_CON_ = *f* (*d* = 40 µm) = 90 s;
- one SG groove machining time: *t*_1groove_ = *t*_ED_ + *t*_ER_ + *t*_CON_ = 343 s;

The key to automize the machining calculation time is in deriving a parametrical equation for feature volume. At fixed input parameters defining BGMs, the groove’s volume calculation becomes a simple geometrical problem.

### 3.2. Micromixer Design for Micro EDM Milling

Altogether, 23 different BGM configurations compliant with micro EDM milling technology were simulated in order to determine their mixing performance. Geometrical parameters, machining times obtained using the technical model and mixing performances determined by simulations are gathered in Table 3.

It can be noted that the highest number of grooves *N*_G_ = 15 can be machined within the time constraint for groove widths between 71 to 106 µm and groove depth of 40 µm. Among the 1-electrode strategy per groove designs (#1-15), the lowest *N*_G_ = 12 is achieved for the narrowest and shallowest of grooves despite removing the smallest volume of material. The reason lies in a very low *MRR* when machining with electrodes of small diameter (e.g., *d*_E_ = 40 µm). When machining the widest and deepest grooves (e.g., #7) also 12 grooves can be machined in nearly the same time despite removing 3.5 times larger volume. Grooves with 2-electrode strategy need significantly more time to be machined.

**Figure 11:**
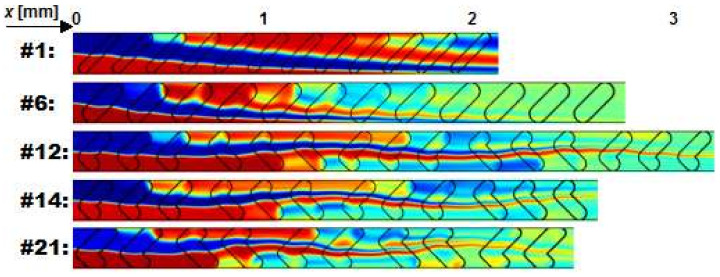
Results of mixing simulations for different BGM designs represented by a concentration surface in the mid-plane of the main channel. Operation conditions are defined by *Re* = 3 and *Pe* = 3670. Green colour denotes complete mixing.

The interpretation of simulated mixing results is further discussed through the following configurations (visualized in Figure 11): #1 as worst performing configuration overall; #6 as best performing configuration overall; #12 as best performing SHM configuration; #14 as second best performing SHM configuration; #21 as best 2-electrode strategy SHM configuration.

Low mixing performance of configuration #1 is mainly a consequence of low number of too narrow grooves, which are inefficient in lateral transportation of fluid. Additionally, it is known from the literature that narrow and shallow grooves in SG configuration perform worse compared to equivalent SH designs (#8-11) [5,15,48]. However, at adequate groove width to depth ratio the results show that in some cases SG outperform SH configurations, as demonstrated by the most efficient design #6 (*CoV* = 0.05, *β*_CoV_ = 1.67). Considering the fixed cross-section of the main channel, this groove design is close to optimal and on the higher end of groove aspect ratios (0.8) investigated in this research. It should be mentioned that SGM realizations reported in the literature were fabricated by soft lithography (e.g., [5,15,49]) and thus, have sharp groove corners. From the perspective of fluid flow pattern, rounded corners seem to induce additional perturbation, which further promotes mixing [36].

SHM configurations #12 and #14 differ in groove depth and number of grooves. Having the same mixing efficiency per groove (*β*_CoV_ = 0.44) shows that groove depth is a critical geometrical parameter. In agreement with assumptions of [6] it is evident that more half-cycles are favourable for all investigated SH configurations (Table 2, #8-23). Similar conclusions as for 1-electrode strategy SHM designs can be made for 2-electrode strategy configurations; namely, deeper grooves and more half-cycles improve mixing performance. The benefit of smaller groove corner roundings is also evident, since configuration #21 renders highest *β*_CoV_ = 0.63 among all SHM designs. This indicates that mixing mechanism in SHMs is impacted by the geometry of SH grooves’ shorter arm.

### 3.3. Simulation Runs of Technical Model

**Figure 12:**
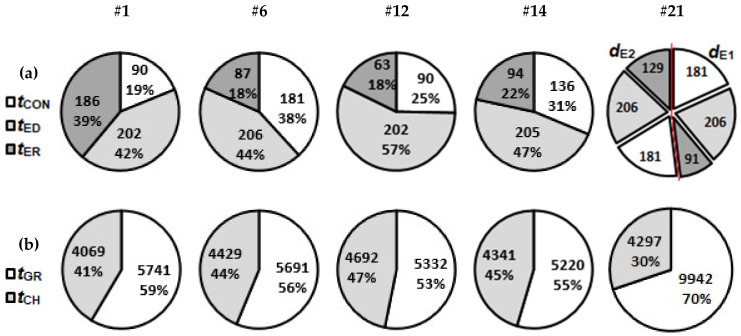
Time required for specific machining operation regarding denoted BGM configurations. (**a**) One groove machining times. For 2-electrode strategy (#21) groove machining times are separately presented with respect to used electrode (*d*_E1_ = 72 µm and *d*_E2_ = 44 µm). (**b**) Machining time of all grooves (*t*_GR_) in a configuration versus corresponding channel machining time (*t*_CH_). Time is given in seconds.

To show the relations between time durations of particular machining operation for BGM geometry configurations highlighted in Section 3.2, relevant pie-charts are presented in Figure 12. Pie-chart for one groove machining of configuration #1 demonstrates the time distribution when using the thinnest electrode (*d*_E_ = 40 µm) considered in this investigation. Electrode dressing and erosion time contribute 81% to overall time and control time due to shallow depth only 19%. Using larger electrode (*d*_E_ = 80 µm) for configuration #6 the share of erosion time significantly drops (18%) due to higher related *MRR*. The electrode control time increases due to bigger depth (38%).

The comparison of one groove machining times for configuration #12 and #14 shows the influence of applying tool electrode of the same diameter (*d*_E_ = 65 µm) to different depths of the grooves. The electrode dressing times are similar (202 s and 205 s respectively), although the share of electrode dressing time *t*_ED_ significantly drops from 57% to 47% on the account of erosion *t*_ER_ and control time *t*_CON_ when machining deeper grooves (#14). Machining operation durations for the 2-electrode strategy are presented through configuration #21. It is evident that, despite machining significantly less volume with the thinner electrode (*d*_E_ = 44 µm, which results in corner rounding of *r*_G_ = 25 µm), it contributes 52% to overall 1 groove machining time.

From the above observations, the following design suggestions related to efficient use of micro EDM milling technology can be drawn. Firstly, for narrow and shallow geometries the *MRR* optimization is crucial for reducing the groove machining times. At wider and deeper grooves efforts to reduce electrode control *t*_CON_ and dressing time *t*_ED_ is the strategy to be pursued (e.g., Figure 12 #6, #14). In this respect, novel electrode wear control strategies such as on-line discharge counting [50], in-situ adaptive control [43] and strategy based on scanning area [41] promise significant reduction of a feature machining duration due to reducing *t*_CON_. 

For low aspect ratio grooves (e.g., Figure 12 #12) the electrode dressing time represents more than half of machining time per groove. Thus, it would be beneficial to reduce *t*_ED_ by optimization of dressing procedure. As for 2-electrode strategies it can be stated that narrow groove corner roundings should be avoided if possible, as usage of smallest electrode diameters dominantly determines the feature machining time.

### 3.4. General Applicability of the Technical Model

The presented technical model is specific to used Sarix micro EDM milling machine tool, manufacturer of which is considered leading in the field. However, some findings can be generalized and are expected to be representative regardless of the type of machine tool used. In this context relations determining *MRR* can be generalized as long as similar discharge energies are applied. Namely, unit material removal per discharge and resulting surface finish chiefly depend on the discharge energy. Thus, for the range of *d*_E_ investigated in this research by applying discharge energies for finishing would result in similar characteristics. By applying the same reasoning, we expect that presented electrode wear characteristics could also be generalized, since again discharge energy level is the dominant influencing parameter. However, as demonstrated by relatively lower electrode wear at thinnest electrode used (e.g., *d*_E_ = 40 µm) less aggressive *regime* reduces the wear effects. On the other hand, the contribution of electrode wear to electrode dressing time, as defined in our investigation, is rather small.

Two of the most common microelectrode dressing procedures are based on the sacrificial block technique and wire EDM grinding (as used in this investigation), the latter being more sophisticated and precise solution. In order to implement sacrificial block technique into the technical model adequate experimental campaign would need to be carried out. We expect that derived relation for *t*_ED_ would significantly differ from relation obtained in this investigation (demonstrated by Equation (4)).

With respect to calculation of *t*_CON_, the linear tool wear compensation method is most commonly implemented on machine tools, thus, similar relations as provided in this investigation are expected. However, duration of ${\bar{t}}_{1\mathrm{CON}}$ may vary between the systems.

Built DKB is also specific to applied workpiece material. We expect that for materials in the steels family the relations should remain similar. This may not be the case when using materials with significantly different thermo-physical properties (e.g., conductive ceramics).

As regards to technical model suitability for wider range of geometries additional exhaustive experimental campaigns should be executed. It is evident that applied machining *regime* significantly depends on the tool electrode diameter, especially if larger *d*_E_ (e.g., *d*_E_ > 0.12 mm) are to be used. Additionally, pocketing strategy could be implemented upon further investigation. At this point it should be stated, that presented technical model usage is limited to groove-like repetitive geometries commonly found in BGM designs. However, the presented technical model framework could be used for the purpose of expanding its applicability by upgrading the supporting DKB. 

## 4. Conclusions

In this paper, a simple empirically based technical model of micro EDM milling is constructed. Its applicability is confined to groove-like microfeatures within a given dimensional interval, but it could be expanded by upgrading the technology DKB using presented methodology. The accuracy of the model lies within 8%, when comparing calculated and measured machining times. It represents an easy to use platform which can be applied for product design optimization and process selection. Demonstration of its usage was presented through optimization of a bottom grooved micromixer (BGM) design. Some general design guidelines particular to micro EDM milling technology are derived:

- When machining narrow slots (*a* < 60 µm) optimization of *MRR* is crucial for reduction of feature machining time.
- At machining wider (*a* > 60 µm) and deeper (*d* > 40 µm) slots reducing electrode control and dressing time is sensible optimization strategy.
- For lower aspect ratio slots (*d*/*a* < 0.6) electrode dressing time contributes dominantly to feature machining time.
- Narrow groove corner roundings should be avoided if possible, despite applying a 2-electrode strategy, due to corresponding very low *MRR*(*d*_E2_).
- Predicted future implementations of concurrent electrode wear estimations could eliminate electrode wear control time and thus, significantly shorten feature machining time. In the case of grooves applied in this investigation from 19% up to 40%.

The mixing performances of 23 micromixers, which are comparable in terms of machining duration, were analysed. Findings are summarised below:

- Investigated BGM designs are readily applicable in metal substrates, thus demonstrating the capabilities of micro EDM milling technology, which in terms of obtainable feature aspect ratios surpasses soft lithography methods.
- The best BGM configuration in terms of mixing performance (*CoV* and *β*_CoV_) was SGM configuration with groove dimension of *a* = 88 µm and *d* = 70 µm, representing a favourable groove geometry.
- SG micromixers with round groove corners achieve better mixing performance. In SH micromixers smaller corner roundings (i.e. sharper corners) perform better.
- More half-cycles for fixed number of grooves improves mixing performance of SH micromixers.
- SGM design is more suitable than SHM for micro EDM milling and gives better mixing performance.
- Lower mixing performances of SHMs may be attributed to reduced effectiveness of the shorter arm due to groove corner roundings which are inherently connected with micro EDM milling technology.

The content of the paper also aims at familiarization of micro EDM milling technology to wider microfluidic research community.

## Figures and Tables

**Table 1:** Geometrical parameters of simulated micromixer designs.

Main Channel Cross-Section (µm)	Groove Inclination Angle	SH Groove Apex Asymmetry	Groove Width *a* (µm)	Groove Depth *d* (µm)	Groove Aspect Ratio	Ridge Length *b* (µm)	Radius of the Groove Corners *r*_G_ (µm)
*w* × *h* = 200 × 50	45°	1/3 × *w*	48–106	40–75	0.38–0.85	70	25–53

**Table 2:** Machining parameters setup particular to Sarix SX-200 EDM milling machine for corresponding *d*_E_.

Parameters	*d*_E_ = 40 µm	*d*_E_ = 50–120 µm	*d*_E_ = 183 µm
*E* (index)	13	13	105
(+,-)	-	-	-
*t*_P_ (indeks)	2	2	6
*f*_P_ (kHz)	180	180	100
*i*_P_ (index)	80	100	65
*u* (V)	77	90	100
*z*_1L_ (µm)	0.7	0.7	0.7
*gain* (index)	100	100	80
*g*_i_ (index)	74	74	80
*n*_CON_	every 10 µm	every 10 µm	every 10 µm

Denotation of parameters: energy index *E*, polarity, duration of discharge pulse *t*_p_, discharge frequency *f*_p_, discharge current *i*_p_, ignition voltage *u*, gain index *gain*, gap index *g*_i_, machining layer depth *z*_1L_, and number of controls of electrode axial wear *n*_CON_.

**Table 3:** Geometrical definition of BGM configurations and simulated mixing performances. Denotations: *r*_G_—groove corner rounding (*a*/2 denotes 1-electrode strategy), *N*_HG_—number of grooves in a half-cycle, *N*_HC_—number of half-cycles, *t*_1groove_—machining time of 1 groove, *t*_GR_—machining time of all grooves in a configuration, *t*_CH_—channel machining time, *t*_TOT_—overall machining time, *CoV*—mixing coefficient of variance , *β*_CoV_—mixing efficiency per groove.

N^o^	Var.	*a* (µm)	*d* (µm)	*r*_G_ (µm)	*N*_GH_	*N*_HC_	*t*_1groove_ (s)	*t*_GR_ (s)	*t*_CH_ (s)	*T*_TOT_ (s)	*CoV*	*β*_CoV_
#1	SGM	48	40	a/2	12	1	478	5741	4069	9810	0.57	0.15
#2	SGM	71	40	a/2	15	1	357	5348	4587	9934	0.15	0.44
#3	SGM	88	40	a/2	15	1	342	5133	4784	9917	0.14	0.48
#4	SGM	106	40	a/2	15	1	335	5019	4981	10000	0.15	0.44
#5	SGM	71	60	a/2	13	1	437	5676	4376	10052	0.07	1.10
#6	SGM	88	70	a/2	12	1	474	5690	4429	10119	0.05	1.67
#7	SGM	106	75	a/2	12	1	467	5603	4587	10189	0.06	1.39
#8	SHM	48	40	a/2	2	6	477	5718	4209	9927	0.32	0.26
#9	SHM	48	40	a/2	3	4	477	5718	4139	9857	0.37	0.23
#10	SHM	48	40	a/2	4	3	477	5718	4104	9822	0.47	0.18
#11	SHM	48	40	a/2	6	2	477	5718	4069	9787	0.47	0.18
#12	SHM	71	40	a/2	3	5	355	5331	4692	10023	0.15	0.44
#13	SHM	71	40	a/2	5	3	355	5331	4622	9953	0.25	0.27
#14	SHM	71	60	a/2	3	4	435	5220	4341	9561	0.19	0.44
#15	SHM	71	60	a/2	6	2	435	5220	4271	9491	0.28	0.30
#16	SHM	88	40	25	3	3	711	6395	4109	10503	0.52	0.21
#17	SHM	88	40	25	2	5	711	7105	4297	11402	0.34	0.29
#18	SHM	106	40	25	3	3	728	6548	4227	10774	0.54	0.21
#19	SHM	106	40	25	2	5	728	7275	4429	11704	0.41	0.24
#20	SHM	88	70	25	3	3	994	8948	4109	13056	0.25	0.44
#21	SHM	88	70	25	2	5	994	9942	4297	14239	0.16	0.63
#22	SHM	106	75	25	3	3	1043	9388	4227	13615	0.26	0.43
#23	SHM	106	75	25	2	5	1043	10431	4429	14860	0.18	0.56
